# Assessment of Knowledge, Attitude, and Practice (KAP) Among Neuro-Critical Care Nurses in Preventing Ocular Surface Disorders in Unconscious, Mechanically Ventilated Patients

**DOI:** 10.7759/cureus.113022

**Published:** 2026-07-20

**Authors:** Narendra Kumar Das, Matuli Das, Lalitha C S

**Affiliations:** 1 Neurological Surgery, Kalinga Institute of Medical Sciences, Bhubaneswar, IND; 2 Ophthalmology, Kalinga Institute of Medical Sciences, Bhubaneswar, IND

**Keywords:** attitude, checklist, eye care, icu, knowledge, mechanically ventilated patients, practice, unconscious

## Abstract

Background: Eyes are vulnerable to exposure and dryness in patients admitted to intensive care units (ICU), leading to significant ocular morbidity in the form of ocular surface disorders. Nurses' knowledge, attitude, and practical skills play a vital role in executing proper nursing care and preventing vision-threatening complications.

Aim: To assess the knowledge, attitudes, and practices (KAP) of eye care measures among neuro-critical care nurses and their relationship in preventing ocular surface disorders in unconscious, mechanically ventilated patients.

Methods: A quasi- experimental pre-post interventional study with a checklist-controlled group was done between August 2022 and July 2024 in a multispecialty tertiary care hospital. Nursing staff working in neuro ICU setups participated in the study, and a validated questionnaire tool was used to collect the data. The association between two categorical variables was measured using the chi-square/Fisher's exact tests.

Results: Among the participants, 64% of them had unsatisfactory knowledge regarding eye care, and 60% had a satisfactory attitude, but only 54% performed satisfactory eye care practices. None of the demographic variables, such as age, experience, or critical care training, had any impact on the practice of eye care. The training on identification, treatment, and preventive strategies, and the use of a checklist on managing ocular surface disorder, significantly improved the performance on all the parameters. Additionally, 80% of the participants demonstrated satisfactory knowledge, satisfactory attitude was noted among 86%, and satisfactory practice performance increased to 82%. The rate of ocular surface disorder reduced from 48% (48) at the beginning of this study to 12% (12) after 12 months post training. The performance was better sustained among the group that consistently used the checklist.

Conclusion: A good attitude, along with adequate knowledge, resulted in appropriate eye care practices. All three domains are interdependent and have a positive relationship with each other. The training on identification, treatment, and prevention strategies, along with the use of a checklist, can significantly lower the rate of occurrence of ocular surface disorder in unconscious, mechanically ventilated patients.

## Introduction

Eyes are vulnerable to dryness and infection in intensive care unit (ICU) patients due to altered normal physiological defense mechanisms, effects of prolonged mechanical ventilation (e.g., decreased venous return leading to chemosis), frequent use of sedatives causing reduction in tear production, incomplete eyelid closure, and environmental factors. If neglected, it can lead to sight-threatening complications, but they are preventable with proper nursing care [1]. Among the many responsibilities of nurses, prioritization of life-saving procedures may lead to neglect in eye care. This could also be attributed to the nurses' lack of knowledge regarding eye care practices [2]. Assessing the understanding and practical skills among nursing staff clarifies the hindrances. Therefore, this study was carried out to assess the knowledge, attitude, and practice (KAP) of eye care measures among nursing staff in ICU setup and understand the relationship of KAP of eye care measures in reducing ocular surface disorders in unconscious, mechanically ventilated patients.

The primary objective was to assess the KAP of eye care measures among neuro-critical care nurses, and the secondary objective was to assess its relationship in reducing ocular surface disorders in unconscious, mechanically ventilated patients.

## Materials and methods

The study population is the nursing staff working in neuro ICU setups during the study period. The nursing staff with a minimum of three months of experience in the ICU and willing to participate were included in the study. The nursing staff who were not present in the ICU at the time of data collection were excluded.

The study commenced after taking approval from the Institutional Ethics Committee (KIIT/KIMS/IEC/705/2021), and the study adhered to the tenets of the Declaration of Helsinki. A total of 116 nursing staff participated in the study. The identification of ocular surface disorder was made on the second and seventh days post admission by use of bedside fluorescein staining of the cornea and viewing with the cobalt blue light of the indirect ophthalmoscope and a 20 Diopter lens. Ocular surface disorders threatening vision, such as corneal abrasions, exposure keratopathy, and microbial keratitis, were identified.

A validated questionnaire tool was used to collect the data. We adopted the tool developed by Saeid et al. [3]. The permission to use the tool was obtained from the author by email. The questionnaire was pre-tested with 10 respondents for its acceptability and consistency. Data from the pilot study were not included in the final analysis. The questionnaire was administered by a one-to-one interaction/interview by one of the authors. The participants were encouraged to clarify any doubts in understanding the questions. The questionnaire contained three sections: A, B, and C. Section A contained seven questions regarding demographic data of the nurses, such as age, gender, qualification, experience in the ICU, type of ICU, information regarding critical care training, and special training in eye care. Section B contained 18 questions to assess knowledge related to the physiology of the eye, the effect of mechanical ventilation, ocular complications, and their management. Section C contained a total of 17 questions; seven questions were related to attitude, and 10 questions were related to the preferred practice. Scores were allotted to questions in Sections B and C. In Section B, it was marked 1 for the correct answer and 0 for the incorrect answer. The total score ranged from 0 to 18. In Section C, the responses to attitude were scored on a 1-5 Likert scale (1 being Very Low and 5 being Very High) [3]. Similarly, practice questions were also scored on a 5-point Likert scale (graded as Always, Often, Occasionally, Rarely, and Never) [3]. The scores of each question under attitude and practice were summed up to get the range of total scores for each domain (attitude score = 7-35 and practice score = 10-50). The total score of each domain was further categorized as "unsatisfactory" if the scores were less than 80% and "satisfactory" if the scores were more than 80% [4,5].

The study, a quasi-experimental pre-post interventional study with a checklist-controlled group, was carried out at a tertiary care multispecialty hospital in Eastern India between August 2022 and July 24. The KAP study design was chosen as it tells us what people know about certain things, how they feel, and how they act [3].

The gap in KAP among the nursing staff who were taking care of unconscious, mechanically ventilated patients was identified and documented. All participating staff underwent training by an ophthalmologist after the gap identification and analysis. The focus of the training was on ocular surface disorders, their possible causes, practice protocols, and prevention strategies.

After the completion of training, each participating staff member was subjected to reassessment on KAP. All the participating staff were divided into two groups of 50 each. The staff from Neuro-ICU1 were designated as Group A, and the staff from Neuro-ICU2 were designated as Group B. Group A provided the care as per their knowledge and skill upgradation during the training session after their gap assessment. Group B provided the care as per their training during the training session after their gap assessment; however, they also used a checklist on risk identification, diagnosis, and prevention. The number of patients who received care during the study period was 100 each in pre- and post-training periods.

The outcome of the study was ascertained by monitoring and comparing the rate of ocular surface disorders before the study commenced and at 12 months after completion of the training program.

Data were collected and entered in Microsoft Excel (version 2007, Microsoft® Corp., Redmond, WA) and further analyzed in Statistical Product and Service Solutions (SPSS, version 27.0; IBM SPSS Statistics for Windows, Armonk, NY). The association between two categorical variables was measured using the chi-square/Fisher's exact tests. The correlation between two continuous variables was assessed using the Spearman correlation test. A p-value less than 0.05 was considered statistically significant.

## Results

In the study, out of the responses of 116 participants, only 100 were included for analysis, and 16 were discarded for being incomplete. Out of these 100, 94% (94) of the study participants were females. The age of the participants was ≤ 25 years (48%, 48) and > 25 years (52%, 52). With respect to experience, 50% (50) of the nurses had more than three years of experience in the ICU. Only 26% (26) of the nurses had received critical care training, and none of the nurses were specially trained for eye care (Table 1).

**Table 1:** General characteristics of the study population (N = 100) Source: Ref [3]

Characteristics/Variables	Frequency (%)
Age	
≤ 25 years	48 (48.0)
> 25 years	52 (52.0)
Gender	
Female	94 (94.0)
Male	6 (6.0)
Experience	
≤ 3 years	50 (50.0)
> 3 years	50 (50.0)
Critical Care Training	
Yes	26 (26.0)
No	74 (74.0)

Less than half of the study participants had "satisfactory" knowledge regarding eye care (36%, 36). The majority of the participants (60%, 60) had a "satisfactory" attitude; however, "unsatisfactory" practice of eye care was observed in 46% (46) (Table 2). Thus, knowledge and the practice of eye care for unconscious, mechanically ventilated patients were found to be deficient in the majority of our study participants.

**Table 2:** Knowledge, attitude, and practice (KAP) scores of study participants before and after training The association was tested using Fisher's exact test or the chi-square test. A p-value of < 0.05 was considered statistically significant. Source: Refs. [3-5]

KAP Domain	Category	Pre-training	Post-training	P-value
Knowledge	Unsatisfactory	64 (64%)	20 (20%)	<0.001 (Chi Sq=39.737)
	Satisfactory	36 (36%)	80 (80%)	
Attitude	Unsatisfactory	40 (40%)	14 (14%)	<0.001 (Chi Sq=17.149)
	Satisfactory	60 (60%)	86 (86%)	
Practice	Unsatisfactory	46 (46%)	18 (18%)	<0.001 (Chi Sq=18.015)
	Satisfactory	54 (54%)	82 (82%)	

In the knowledge domain, the performance was found to be better in the group where the age was ≤ 25 years and among participants with critical care experience (p < 0.001). The study participant with longer duration of experience performed better in their attitude scores (p < 0.005). None of the demographic variables demonstrated any relationship with the practice of eye care (Table 3).

**Table 3:** Association of knowledge, attitude, and practice with general characteristics of the study population (N = 100) The association was tested using Fisher's exact test or the chi-square test. A p-value of < 0.05 was considered statistically significant. Source: Refs. [3-5]

Parameters	Knowledge (Unsatisfactory)	Knowledge (Satisfactory)	Attitude (Unsatisfactory)	Attitude (Satisfactory)	Practice (Unsatisfactory)	Practice (Satisfactory)
Age ≤25 years	28 (51.8)	20 (55.6)	22 (55.0)	26 (43.4)	26 (52.0)	22 (44.0)
Age >25 years	36 (49.2)	16 (44.4)	18 (45.0)	34 (56.6)	24 (48.0)	28 (46.0)
P value	0.256 (Chi Sq=1.287)		0.252 (Chi Sq=1.309)		0.423 (Chi Sq=0.641)	
Female	60 (93.7)	34 (94.4)	38 (95.0)	56 (93.3)	44 (95.6)	50 (92.5)
Male	4 (6.3)	2 (5.6)	2 (5.0)	4 (6.7)	2 (4.4)	4 (7.5)
P value	0.888 (Chi Sq=0.174)		0.731 (Chi Sq=0.118)		0.520 (Chi Sq=0.412)	
≤ 3 years exp	31 (48.5)	19 (52.7)	18 (75)	32 (42.1)	24 (57.1)	26 (44.8)
> 3 years exp	33 (51.5)	17 (47.3)	6 (25)	44 (57.9)	18 (42.9)	32 (55.2)
P value	0.676 (Chi Sq=0.174		0.005 (Chi Sq=7.895)		0.224 (Chi Sq=1.478)	
Training Yes	10 (15.6)	16 (36.7)	10 (33.3)	16 (22.8)	11 (22.4)	15 (27.2)
Training No	54 (84.4)	20 (63.3)	20 (66.7)	54 (77.2)	34 (77.6)	40 (72.8)
P value	0.001 (Chi Sq=9.946)		0.273 (Chi Sq=1.198)		0.748 (Chi Sq=0.103)	

The scores at one year after the training on identification, treatment, and prevention of ocular surface disorders showed significant improvement in all the components. The knowledge score improved to "satisfactory" levels among 80% of the study participants (p < 0.001). Similarly, 86% (p < 0.001) of the study participants showed "satisfactory" attitude towards eye care, while 82% (p < 0.001) demonstrated "satisfactory" practice skills (Table 2).

The performance of Group B staff was much superior in knowledge (p < 0.001), attitude (p < 0.05), and practice (p < 0.001) as compared to Group A staff, who did not use the checklist, at 12 months post training (Table 4).

**Table 4:** Knowledge, attitude, and practice (KAP) scores of study participants at 12 months in Group A and Group B The association was tested using Fisher's exact test or the chi-square test. A p-value of < 0.05 was considered statistically significant. Group A: Checklist not used; Group B: Checklist used. Sources: Refs. [3-5]

KAP	Group A	Group B	P-value
Knowledge: (Unsatisfactory)	28 (28%)	10 (10%)	0.001 (Chi Sq=10.526)
Knowledge: (Satisfactory)	72 (72%)	90 (90%)	
Attitude: (Unsatisfactory)	18 (18%)	8 (8%)	0.035 (Chi Sq=4.421)
Attitude: (Satisfactory)	82 (82%)	92 (92%)	
Practice: (Unsatisfactory)	24 (24%)	6 (6%)	0.0004 (Chi Sq=12.706)
Practice: (Satisfactory)	76 (76%)	94 (94%)	

Correlation analysis suggested a positive correlation of knowledge scores with attitude scores (r = 0.22) and practice scores (r = 0.52) at one year post training.

The rate of ocular surface disorder among unconscious, mechanically ventilated patients was 46% (46) before the commencement of this study. The rate improved to 12% (12) at one year after training; however, the rate was at 4% (4) in Group B, where the staff used the checklist, and 8% (8) in Group A, where the staff had received the training but did not use the checklist in everyday practice (Table 5).

**Table 5:** Rate of ocular surface disorders before the training and at one year post training Group A: Checklist was not used, Group B: Checklist was used.

Rate at the Beginning of the Study	Rate at 1 Year (Post Gap Analysis & Training)	Rate at 1 Year (Group A)	Rate at 1 Year (Group B)
46% (46)	12% (12)	8% (8)	4% (4)

## Discussion

The key challenges faced by critically ill, unconscious, sedated patients on ventilatory support are their need for prolonged hospital stay, vulnerability to healthcare-associated infections [6,7], total dependence on ICU nursing staff who are mainly focused on life-threatening problems, and eye care does not get the priority. Therefore, ICU nursing staff who are trained in providing ophthalmic are becomes vital [8]. In general, most of the eye care services are provided on an outpatient or day care basis and are assisted by trained ophthalmic assistants [9,10].

On analyzing the responses in the knowledge domain at the commencement of our study, we found that nurses had good knowledge on some aspects of eye care, but, on the whole, the performance was unsatisfactory for the majority of our study participants. This inadequacy in knowledge may be the reason for the high rate of ocular surface disorder in our patients. There was a lack of awareness of assessing general appearance and protective eye mechanisms in ICU patients. A few of the participants knew about the negative effects of sedatives and muscle relaxants on protective physiological mechanisms of eye blinking. Knowledge gaps were also noted regarding proper lid closure and application of lubricating eye drops or ointments, the proper way of eye cleaning, the appropriate size of eye pad or cover to be used, and preventive measures for various grades of lagophthalmos. Similar findings were noted in a study by Güler et al. [11].

Knowledge was found lacking regarding the potential risk factors, such as ICU temperatures and the effects of tightly securing the endotracheal tube, in causing eye disorders. Most of them thought that only following sterile techniques while performing endotracheal suctioning prevents eye complications. However, a few of the participants acknowledged the need to cover the patient's eye and perform suctioning from the side of the bed. In a study by Milutinovic et al. [12], only 17% of the nurses were aware of the proper way of suctioning. Twenty-five percent of our nurses also knew the use of polyethylene cover as the best preventive method for corneal abrasion, which was better than what was documented by Milutinovic et al. [12]. This preventive measure has also been highlighted in a randomized controlled study by So et al. [13]. Very few nurses were aware of the best time to begin and administer eye care in ICU patients, as time plays a critical role in the onset of dryness and exposure keratopathy, with the maximum incidence being documented from five days to two weeks [14,15].

The level of knowledge of our nurses was comparable to what was documented in the studies by Milutinovic et al. and Cho et al. [12,16]. In a study by Alghamdi et al. [17], 22.2% of nurses in the surgical ICU reported "satisfactory" knowledge. Our critical care-trained nurses also exhibited "satisfactory" knowledge, which was found significant (p < 0.001) as compared to the participants who had no exposure to critical care training. In our study, the years of work experience and basic qualification did not have any impact on knowledge.

The majority of the study participants had a "satisfactory" attitude towards eye care practices in the ICU in terms of practicing infection control measures. None of the demographic variables were significantly associated with a "satisfactory" attitude score, except for the group of study participants who had more than three years of working experience (p < 0.005). Milutinovic et al. [12] noted that male gender and working experience of more than 10 years had a more positive impact on nurses' attitudes. Perhaps the years of experience expose the nursing staff to observe the consequences of preventable ocular surface disorder, thus improving their attitude towards eye care.

The total practice score in our study was similar to what has been documented by other studies [12]. The demographic variables, such as age, gender, experience, or critical care training, did not have any impact on the practice scores of the participants. The possible reason may be the lack of specific training on eye care protocols.

To sum up the findings of our study, none of the three domains demonstrated a significant association with any of the demographic variables. The current literature data also show similar findings [17,18]. Multiple studies have also demonstrated that there was no statistically significant correlation between knowledge about preventive eye care measures and their attitudes or nursing practices [17-19]. However, we observed a positive correlation between knowledge and attitude scores (r = 0.22) and between knowledge and practice scores of the nurses (r = 0.52). Similar observations were also captured in a study by Khalil et al. [4].

The gap analysis allowed us insight into the deficiencies and training needs. After the training on identification, treatment, and preventive strategies, the participants demonstrated improvement in all three components of KAP, which was statistically significant. However, the improvement was better sustained in the group that used the checklist consistently. The use of a checklist ensures repeated reinforcement of the workflow, thus resulting in better sustained outcomes. The use of a checklist in reducing the incidence of ocular surface disorder among unconscious patients on prolonged ventilation has not been documented in earlier studies.

Some researchers consider that the lack of knowledge, attitude, and skill should be considered as a barrier to providing eye care, and improving them to a sufficient level plays a key role in providing high-quality care [20,21]. Knowledge and attitude also affect professional behavior as they have an impact on nursing practices and interventions. Pourghaffari Lahiji et al. [22] have pointed out that it is necessary to intensify eye care education aimed at inexperienced nurses and provide continuing education on the latest eye care methods to experienced nurses. The current study indicates that the staff with critical care training performed better, and deficiencies in knowledge and practice were noted among both experienced and inexperienced staff. Therefore, the need for training on identification, treatment, and preventive strategies cannot be overemphasized. The present study also demonstrates that improvement in knowledge leads to better attitude and practice skills. The use of a checklist was found to be a useful tool in sustaining knowledge and practice standards.

The effectiveness of the training program was well demonstrated in the current study. The training can also help them in expanding their role in early diagnosis, such as detecting exposure keratopathy and assessing the ocular surface [20]. However, the major challenges are sustainability in maintaining the knowledge base and quality standards over a long-term period. This can be overcome by regular use of a checklist, as noted in our study, by conducting regular clinical audits, regular training on identification, treatment, and preventive strategies for ocular surface disorder and incorporating eye care protocols in the ICU care manuals.

The previous studies have shown that adherence to evidence-based practices and use of standardized operating procedures (SOPs) improve the eye care protocols [20,22]. Our study has clearly shown that training of the staff and using a simple checklist for eye care led to significant improvement in reducing the ocular surface disorder among unconscious, mechanically ventilated patients.

Limitations

Our sample size was small, and the study was limited to a single center. Further studies may be useful with a larger sample size and randomization of the participants. Having a few open-ended questions might have provided deeper insight into knowledge and practice gaps to guide future training needs.

## Conclusions

A positive attitude, along with appropriate knowledge, resulted in satisfactory eye care outcomes. All three domains are interdependent and have a positive relationship with each other. Enhancing nurses' knowledge through a structured training program, the use of checklists, and regular clinical audits will improve and sustain eye care practices and outcomes of ocular surface disorder in unconscious, mechanically ventilated patients.

## Appendices

Validated questionnaire tool 

SECTION: A (Demographic Data)

1: Age

2: Gender

3: Qualification

4: Experience in ICU

5: Type of ICU

6: Critical care training

7: Special training in eye care

SECTION: B (Knowledge Domain)

1) Which of the following factors disturbs the blink reflex?

A) Use of sedative agents and muscle relaxants

B) Simultaneous use of eye drops and ointmentsC) Use of eye pads

D) Inadequate nutrition and electrolyte imbalance

E) I don’t know

2) Which of the following choices is a potential risk factor for eye disorders?

A) Ward temperature, tightly securing the endotracheal and tracheostomy tube

B) Failure to maintain sterility during endotracheal suctioning

C) Use of adhesive tape for closing the patient’s eyes

D) None

E) I don’t know

3) What is the most important criterion in assessing eye disorders in the ICU?

A) Funduscopic examination

B) General appearance of the eye and its protective mechanisms

C) Patient’s hemodynamic status

D) History of eye surgery

E) I don’t know

4) Which factors aggravate chemosis?

A) Tightly securing the endotracheal tube

B) Pain medication overdose

C) Prolonged hospitalization

D) High blood pressure

E) I don’t know

5) The best time for beginning and administering eye care for patients hospitalized in the ICU is

A) Immediately after hospitalization

B) Within the first two to seven days of hospitalization

C) From the second week of hospitalization on

D) As needed (PRN)

E) I don’t know

6) How often should the patient be assessed regarding the protective mechanisms of the eye (ability to blink, etc.)?

1) On a daily basis

2) In every working shift

3) On a weekly basis

4) Twice a week

5) I don’t know

7) How should endotracheal suctioning be performed to prevent eye complications?

A) Merely by using sterile technique

B) Performing suctioning from the side of the bed while covering the patient’s eyes

C) Performing suctioning as short as possible while standing above the patient’s bed

D) No difference

E) I don’t know

8) What is the proper way for cleansing a patient’s eyes?

A) From the inner canthus outward

B) Cleansing each eye separately

C) Only the use of sterile technique, not the direction, is important

D) It is totally different when an infection is present

E) I don’t know

9) What is the appropriate size for eye pads and covers?

A) Exactly as big as the eye socket

B) Covering from the top of the eyebrow to the bottom of the eye socket

C) Covering about four centimeters above and below the eye socket

D) Size does not matter

 E) Don’t know

10) How should eye care be provided for a patient who can blink and close his eyes completely?

A) This patient does not need eye care

B) Every two hours and by using eye lubricants

C) A mere use of supplementary drops such as artificial tears

D) Every two hours and closing the eyes by using adhesive tape once the patient is asleep

E) I don’t know

11) What is the best eye care for a patient who cannot close his eyes and his sclera is exposed?

A) Only closing his eyes by using adhesive tape

B) Cleansing his eyes by using normal saline every four hours and administrating eye lubricants

C) Administrating eye lubricants, closing his eyes, and using appropriate eye drops every two hours

D) Administrating topical antibiotics and cleansing the eyes every four hours

E) I don’t know

12) Who should provide eye care for a patient who is unable to blink and, hence, his sclera and pupil are exposed?

A) Cleansing the eyes by normal saline on an hourly basis

B) Administrating eye lubricants, closing his eyes, and administrating simple eye ointment every four hours

C) Administrating appropriate antibiotic drop as ordered by the attending physician

D) Merely closing the eyes by using adhesive tape while excluding eye cleansing

E) I don’t know

13) How should eye care be given to a patient who receives mechanical ventilation and sedative agents?

A) No type of care is needed

B) The initial eye cleansing as well as closing the eyes suffices

C) Closing the eyes and using eye lubricants and ointments every four hours

D) Providing eye care every two hours and contraindicating PEEP mode

E) I don’t know

14) The key objective of eye care is

A) Preserving corneal integrity

B) Preventing eye infections

C) Preventing Chemosis

D) Providing simple routine care

E) I don’t know

15) The best eye care plan is

A) Every two hours only and in the first week of hospitalization

B) As needed and as ordered by the physician

C) Use of a fixed care plan for all patients

D) Effective staff education and the use of appropriate clinical algorithms

E) I don’t know

16) Which of the following methods is the most effective for preventing corneal abrasion?

A) Use of polyethylene eye covers

B) Use of eye drops and ointments

C) Mechanical closure of the eyes by using adhesive tape

D) Eye cleansing by normal saline

E) I don’t know

17) Eye cleansing by normal saline

A) Humidifies the eyes and suffices

B) Is not recommended on its own because it can bring about eye dryness

C) Is not recommended alongside all other eye lubricants

D) Is recommended only for conscious patients

E) I don’t know

18) What is the right direction for applying adhesive tape on eyelids for closing the eyes?

A) Vertical

B) Horizontal

D) Direction is not important

C) Oblique

D) I don’t know

SECTION: C (Attitude Domain)

1. How much effect does pre- and post-procedure hand washing have of preventing or reducing the incidence of eye disorders?

2. How much importance does eye care have for patients receiving mechanical ventilation?

3. How much priority do you give to eye care in patients receiving mechanical ventilation?

4. How much willingness do you have to provide eye care for patients receiving mechanical ventilation?

5. How much effect does staff education in terms of eye care have on preventing eye disorders?

6. How much effect does eye care performed every four hours have on preventing eye disorders?

7. How much effect does standard endotracheal suctioning have on reducing the incidence of eye disorders?

Type of Care: (Practice Domain)

1. Washing hands before and after procedures

2. Assessing and ensuring that the patient’s eyes are closed (on a daily basis)

3. Assessing and ensuring that the endotracheal or tracheostomy tube is correctly fixed in place (on a daily basis )

4. Performing suctioning while standing beside, not above, the patient’s bed and covering the patient’s eyes (PRN)

5. Cleaning the eyes by using normal saline or sterile water every two hours

6. Administrating appropriate eye lubricants every two hours

7. Administrating appropriate eye drop every two hours

8. Administrating appropriate eye ointment every four hours

9. Caring for each eye separately in case of unilateral eye infection

10. Using adhesive tape for closing the patient’s eyes appropriately

The permission to use the tool was obtained from Saeid et al. [3] by email.
